# fDETECT webserver: fast predictor of propensity for protein production, purification, and crystallization

**DOI:** 10.1186/s12859-017-1995-z

**Published:** 2018-01-03

**Authors:** Fanchi Meng, Chen Wang, Lukasz Kurgan

**Affiliations:** 1 Department of Electrical and Computer Engineering, University of Alberta, Edmonton, AB Canada; 2 Department of Computer Science, Virginia Commonwealth University, Richmond, VA USA

**Keywords:** X-ray crystallography, Protein production, Protein structure determination, Target selection, Structural genomics, Prediction

## Abstract

**Background:**

Development of predictors of propensity of protein sequences for successful crystallization has been actively pursued for over a decade. A few novel methods that expanded the scope of these predictions to address additional steps of protein production and structure determination pipelines were released in recent years. The predictive performance of the current methods is modest. This is because the only input that they use is the protein sequence and since the experimental annotations of these data might be inconsistent given that they were collected across many laboratories and centers. However, even these modest levels of predictive quality are still practical compared to the reported low success rates of crystallization, which are below 10%. We focus on another important aspect related to a high computational cost of running the predictors that offer the expanded scope.

**Results:**

We introduce a novel fDETECT webserver that provides very fast and modestly accurate predictions of the success of protein production, purification, crystallization, and structure determination. Empirical tests on two datasets demonstrate that fDETECT is more accurate than the only other similarly fast method, and similarly accurate and three orders of magnitude faster than the currently most accurate predictors. Our method predicts a single protein in about 120 milliseconds and needs less than an hour to generate the four predictions for an entire human proteome. Moreover, we empirically show that fDETECT secures similar levels of predictive performance when compared with four representative methods that only predict success of crystallization, while it also provides the other three predictions. A webserver that implements fDETECT is available at http://biomine.cs.vcu.edu/servers/fDETECT/.

**Conclusions:**

fDETECT is a computational tool that supports target selection for protein production and X-ray crystallography-based structure determination. It offers predictive quality that matches or exceeds other state-of-the-art tools and is especially suitable for the analysis of large protein sets.

## Background

X-ray crystallography is the dominant method to derive protein structures. It was used to produce slightly over 90% of the currently available structures [1, 2] [source: www.rcsb.org]. However, these efforts suffer relatively low success rates ranging between 2 and 10% [3–5]. The low rates stem from cumulative attrition along the protein production and crystallization pipelines. The unsuccessful attempts were shown to account for over 60% of the structure determination costs [6, 7]. One of solutions is to select protein targets that are amenable to the diffraction-quality crystallization. Target selection benefits from computational methods that estimate propensity of proteins for the completion of various steps of the X-ray crystallography-based structure determination pipelines [8].

The drawbacks related to the high attrition rates were actively investigated over the last two decades. Data coming from the protein production and structure determination experiments, which are available in databases such as TargetTrack [9, 10] and PepcDB [11], were used to derive protein sequence-derived markers of amenability of proteins to the production and structure determination [12–19]. These results motivated the development and use of sequence-based target selection tools. These tools predict propensity for protein production and structure determination directly from the protein sequences. While majority of them are focused on the prediction of propensity for the final, structure production step [5, 20–22], a few tools that offer a broader scope were developed recently. The first such tool, PPCpred [23], addresses prediction of success of the protein production, purification, crystallization, and diffraction-quality crystallization (the final structure determination step). Two other similar in scope tools were published in the last three years: PredPPCrys [24] and Crysalis [25]. However, PPCpred and PredPPCrys require a substantial amount of computations and consequently they take a relatively long time to produce results. Our aim is to provide a fast webserver for the comprehensive prediction of the four steps of the crystallization pipeline that rivals accuracy of the two slow methods and outperforms the fast Crysalis on the predictive quality.

The ability to make fast predictions is important for a number of reasons. One application is to facilitate studies that aim to increase structural coverage of the protein sequence space. In this context, fast methods should be used for the selection of favorable targets, in terms of their propensity for successful production and structural determination, from large and structurally uncharacterized protein domain families, and from structurally uncharacterized subfamilies in very large and diverse protein families that have incomplete structural coverage [26–28]. This involves analysis of hundreds or thousands of proteins at the time to find close homologs that are more likely to crystallize [29]. Another vital application of the computationally efficient predictors addresses estimation and analysis of attainable structural coverage of specific organisms and taxa [30], which is of substantial interest to pharmaceutical research [31, 32].

We originally designed the fDETECT (fast Determination of Eligibility of TargEts for CrysTallization) method [30] to rapidly predict propensity of the protein sequences for the diffraction-quality crystallization. The implementation of the original version of fDETECT was never made available and the algorithm itself covers only the last step of the crystallization pipeline. Using the datasets and design protocols which we utilized to design the original predictive model, we extended our tool to cover the four steps without the loss of speed. We are also making it available as a convenient to use webserver that can be found at http://biomine.cs.vcu.edu/servers/fDETECT/.

## Methods

### Datasets

We designed fDETECT using the training dataset from ref. [23]. This dataset includes 3587 proteins collected in 2010 from the PepcDB database [11]. They were annotated based on the corresponding stop status and current status fields. We utilized the “sequencing failed”, “cloning failed” and “expression failed” stop statuses to define success of the protein production step. We did not consider a separate prediction of the success of cloning since this step is characterized by very high, nearly 100% success rates [33–35]. We used the “purification failed” stop status to define the success of the purification step, and “crystallization failed” and “poor diffraction” stop statuses for the crystallization step. Finally, we annotated proteins for which the diffraction-quality crystallization step is successful based on their “structure successful”, “TargetDB duplicate target found” and “PDB duplicate found” stop statuses, as well as the “crystal structure” and “in PDB” current statuses. As it is assumed for the other predictors in this area, we map the protein sequences to these four outcomes without considering inter-molecular characteristics of the crystallization process, such as use of specific tags or buffers. We removed duplicate sequences with different outcomes by deleting the trials with an earlier stop status. Finally, using BLASTCLUST we reduced the sequence identity among chains that belong to the same protein production and crystallization step to below 25%. This is consistent with the threshold used in related studies [23, 24, 36, 37]. The same source database and similar protocol to collect and annotate the crystallization trials were used to design the PPCpred [23], PredPPCrys [24] and Crysalis [25] methods.

We established two new test datasets to evaluate and compare predictive quality of fDETECT and the other predictors. We collected the source data from the TargetTrack database [9, 10] (http://sbkb.org/), which supersedes the PepcDB database, in November 2016. We selected proteins that correspond to the four predictions generated by fDETECT: failure of material production (MF), failure to purify (PF), failure to crystallize (CF) and success to yield diffraction-quality crystals (CR). The selection makes use of the following trial stop statuses from the TargetTrack:

- MF: sequencing failed; cloning failed; expression failed
- PF: purification failed
- CF: crystallization failed; poor diffraction
- CR: structure successful; PDB duplication found

This approach is in agreement with the annotations used to derive other relevant methods [23–25, 30].

In total, we found 35,705 MF trials, 5823 PF trials, 2582 CF trials and 2012 CR trials. We deleted sequences shorter than 30 residues, which correspond to peptides, and sequences that contain non-standard amino acids. We clustered the remaining proteins using Blastclust to find and remove identical chains (-S 100 -L 1 parameters). For every pair of identical chains, we kept only the one that made it to the step farthest into the crystallization process. For example, if a protein sequence with the MF status was also found to have PF status then we removed its MF status since apparently material production has succeeded. There were 33,317 MF sequences, 5631 PF sequences, 2560 CF sequences and 2004 CR sequences after we applied this filtration. Next, we reduced similarity between this dataset and the training dataset to include only the proteins that are at most 25% similar to any of the training proteins. To accomplish that we clustered the combined set of the remaining proteins and training proteins using Blastclust (-S 25 –L 0.9 parameters) and we retained only the clusters that do not include any of the training proteins. Consequently, the resulting set of test proteins that share <25% similarity to the training proteins includes 22,243 MF proteins, 3468 PF proteins, 1213 CF proteins and 980 CR proteins. We used the entire set of CR proteins and we sampled at random the same number of equally divided MF, PF and CF proteins. This corresponds to 327 MF, 327 PF, 327 CF, and 980 CR proteins, for the total of 1961 sequences that make up the TESTlarge dataset. Moreover, we devised the TESTsmall dataset with 432 sequences by randomly sampling 72 MF, 72 PF, 72 CF and 3*72 = 216 CR proteins from the TESTlarge dataset. TESTsmall is necessary to accommodate for the relatively heavy computational cost of running some of the predictors that are included in our comparative analysis, in particular PPCpred [23] and PredPPCrys [24]. The heavy computational cost is compounded by the inclusion of a large set of seven methods in our comparative analysis. Both TESTlarge and TESTsmall datasets have the 50/50 balanced split of proteins that were solved structurally via crystallization and for which the structure determination efforts failed. This type of split facilitates a reliable empirical comparison with representative methods that predict only the propensity for the diffraction-quality crystallization. Use of an unbalanced dataset may lead to skewed measurements of predictive performance. To reiterate, both test datasets include proteins that share <25%, similarity to the training dataset of fDETECT. They are available on the fDETECT webserver page at http://biomine.cs.vcu.edu/servers/fDETECT/.

### Evaluation

We designed the fDETECT using only the training dataset. Once the design was completed, we tested this model on the TESTlarge and TESTsmall datasets. We used the same test datasets to compare fDETECT with the three other methods that cover multiple steps of the crystallization pipeline: PPCpred [23], PredPPCrys [24] and Crysalis [25]. We also compared fDETECT to a set of four representative webservers that focus solely on the prediction of the diffraction-quality crystallization: XtalPred [7, 38], CRYSTALP2 [39], XtalPred-RF [40] and TargetCrys [37]. Similar to the other relevant studies [23–25, 37, 39, 40], we assessed predictive quality using MCC, accuracy and AUC measures. The first two measures are used to evaluate binary predictions, i.e., a given step is predicted to fail vs. to succeed. The AUC, which quantifies the area under the ROC curve, is used to assess predictive quality of real-valued propensities generated by these predictors.

### Design of the predictive model

We used a machine learning approach inspired by the design of the original version of the fDETECT method to design the new fDETECT webserver. The webserver works in two steps. First, the input amino acid sequence is converted into four fixed-sized vectors composed of empirically selected numerical features that represent various physicochemical characteristics of the corresponding protein. Second, these four feature vectors are input into the corresponding four logistic regression models to generate predictions for the MF, PF, CF, and CR proteins. The regression was selected based on its favorable predictive performance and a significantly lower runtime when compared against several other types of classifiers including Support Vector Machine with linear, polynomial, RBF and sigmoid kernels, and Gaussian radial basis function network; these results are included in ref. [23].

We considered a comprehensive set of 1276 features that were computed based on:

- Amino acid composition: 420 features including 20 single amino acids and 400 dipeptides.
- Clusters of amino acid types: 336 features that divide amino acids into groups based on their physicochemical properties, such as hydrophobicity, van der Waals volume, polarity, polarizability, charge, secondary structure, and solvent accessibility to compute composition, transition, distribution, and characteristics of residue segments that have these properties.
- Physicochemical properties of individual amino acids: 448 features based on average (per sequence), minimal and maximal (per sequence segment) values of 64 hydrophobicity and energy based indices collected from the AAIndex database [41].
- Physicochemical properties of proteins: 4 features that include the isoelectric point, aliphatic index, instability index and charge of the input protein sequence.
- Sequence complexity and putative intrinsic disorder of the input protein chain: 68 features that were derived from the predictions of disorder generated with IUpred [42] and the sequence complexity produced with the SEG algorithm [43].

A detailed description of these features is available in the supplement from ref. [23].

Next, the considered features were subjected to an empirical selection to find small subsets that are relevant to the prediction of each of the four outcomes. The selection process included two steps: 1) removal of irrelevant and mutually similar features; and 2) wrapper-based selection to maximize predictive performance. Both steps were performed using five-fold cross-validation on the training dataset. In the first step, the 1276 features were reduced to remove those that have weak correlation with the predictive outcome (biserial correlation with the annotation of the outcomes <2*average biserial correlation of all considered features) and which are mutually correlated (Pearson correlation >0.7). The remaining features were used in the second step with the logistic regression predictor to select a subset of features that maximize value of AUC on the training dataset. We initialized the set of selected features with the feature that has the highest biserial correlation and we attempted to add each of the subsequently ranked features to the selected set of features. We added a given feature if this addition resulted in an improved value of the AUC score; otherwise the feature was rejected. Consequently, the original set of 1276 was reduced to 9, 8, 4, and 11 features for the prediction of the MF, PF, CF and CR steps, respectively.

We summarized the breakdown of these four features sets in Table 1. Interestingly, each of the five major groups of features is used to predict at least two of the outcomes and each outcome utilizes a different breakdown of the number of features between the feature groups. This justifies our approach to select a different set of features for each of the four outcomes. It also points to a distinct nature of the relation between the input protein chains and the success of a given protein production and crystallization step. We used a similar approach in the design of our accurate but much slower PPCpred method [23]. The main differences between these two methods are that for fDETECT: 1) we used a much faster to compute features, in particular those that do not require calculation of the computationally demanding sequence alignment; 2) we utilized a larger set of more diverse and sophisticated features (1276 features for fDETECT vs. 828 for PPCpred); and 3) we applied a much faster to compute predictive model (regression for fDETECT vs support vector machine for PPCpred). Altogether, this resulted in a substantially faster to compute prediction.

**Table 1:** Summary of the considered and selected feature sets

Feature types	The complete set of considered features	Features used to predict MF step	Features used to predict PF step	Features used to predict CF step	Features used to predict CR step
Amino acid composition	420	2	2	0	3
Clusters of amino acids types	336	2	3	1	1
Physicochemical properties of amino acids	448	3	2	2	5
Physicochemical properties of proteins	4	1	1	1	1
Sequence complexity and intrinsic disorder	68	1	0	0	1
Total	1276	9	8	4	11

## Results

### Webserver

The fDETECT’s webserver is freely available at http://biomine.cs.vcu.edu/servers/fDETECT/. The computations are performed on the server side. Users needs a modern web browser (Firefox, Chrome, or Internet Explorer) and an internet connection to process the predictions. To use our service, a user is asked to input protein sequence(s) in FASTA format and an e-mail address that is used to notify the user when the results are ready and where to find them. The results are also delivered in the web browser window. The fDETECT webserver allows for batch prediction of up to 1000 protein sequences. It also offers an option to run the PPCpred method, which due to a substantial computational cost is limited to runs for up to 5 proteins.

**Fig. 1:**
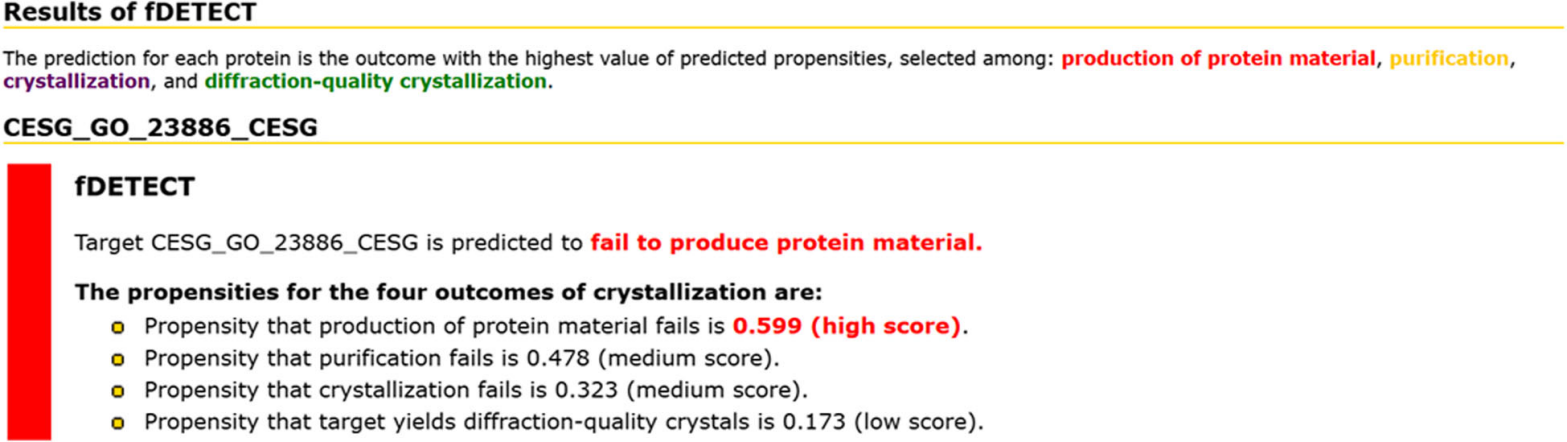
Sample result generated by the fDETECT webserver

The webserver outputs results in the HTML format and in downloadable text-based format. For the user’s convenience, we provided code written in Python to parse the text-based output. The results include predicted numeric propensities for the four outcomes and an overall prediction based on the highest propensity. The HTML page, see Fig. 1, visualizes the results using user-friendly scheme that includes color-coded predictions where red corresponds to the MF prediction, yellow to PF, purple to CF, and green to CR. This page also provides interpretation of the putative propensities that are associated with each prediction using three confidence levels: low, medium and high. These levels were determined by analyzing the propensities generated for each experimentally determined outcome (MF, PF, CF and CR) on the training dataset. The “low”/"high” category corresponds to the lowest/highest 20% of the propensities and “medium” is for the remaining propensities between 20 and 80 percentiles. Detailed instructions how to process and interpret a prediction are available on the “Tutorial and Help” page on the website of the webserver.

### Comparative evaluation of runtime

**Fig. 2:**
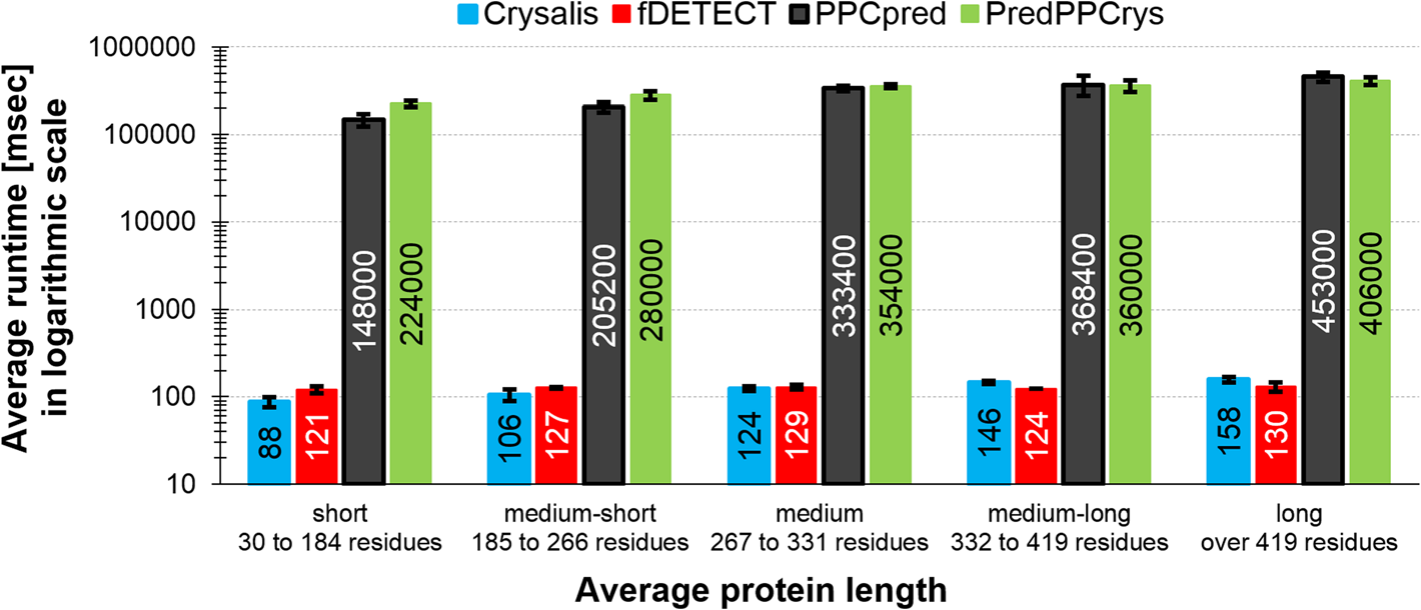
Comparative analysis of runtime. The analysis covers the four methods that predict the four steps of the protein production and crystallization process. Proteins in the TESTsmall dataset were divided into five equally sized subsets with increasing sequence length (very short, short, medium, long and very long). For each subsets of proteins and each of the four predictors we show the average runtime [msec] as bars, the numerical value of the average inside the bars, and the corresponding standard deviation as the error bars

We compared the runtime of the four methods that predict the four steps of the protein production and crystallization process (fDETECT, PPCpred, Crysalis and PredPPCrys) in Fig. 2. We also investigated whether their runtime varies with the sequence length. To accomplish that, we divided the proteins in the TESTsmall dataset into five equally sized subsets with increasing sequence length (short, medium-short, medium, medium-long, and long) and measured the average runtime for each subset. The results reveal that two methods, fDETECT and Crysalis, perform prediction three orders of magnitude faster than the other two methods, PPCpred and PredPPCrys. On average, both fast methods predict a protein sequence in about 0.1 s while the other two methods take about 5 min for the same prediction. We found that the fDETECT’s runtime does not increase as the length of the proteins grows. The runtime of the other three methods grows with the sequence length. For instance, the ratio of the average runtime between long and short protein sets equals 1.08, 1.80, 3.06 and 1.81 for fDETECT, Crysalis, PPCpred and PredPPCrys, respectively. To compare, the median length of short proteins is 138 residues while the median length of the long proteins is 3.8 times larger and equals 523 residues.

We performed linear fitting of the measured runtime as a function of protein chain length. We summarized these results in Table 2. Our empirical results reveal that this linear approximation provides an accurate estimate of the total runtime on the TESTsmall dataset. For instance, we estimated the total runtime for fDETECT to be 0.90 min vs. the measured runtime of 0.88 min (2.3% error) and 27.49 h vs 28.35 h for PPCpred (3% error). We used this linear approximation to estimate the expected runtime to predict proteins in the whole human proteome. This runtime equals about 0.7 h for both fDETECT and Crysalis vs. 76 and 79 days for PPCpred and PredPPCrys, respectively. This demonstrates that fDETECT is significantly faster than PPCpred and PredPPCrys methods, and that it can be easily used to analyze whole proteome-size datasets.

**Table 2:** Comparison of estimated and measured runtime

Predictor	Coefficients of the linear fit into the measured runtime values	Runtime estimated using linear fit for the human proteome # sequences = 20,193 average length = 561	Runtime estimated using linear fit for the TESTsmall dataset # sequences = 432 average length = 332	Runtime measured for the TESTsmall dataset # sequences = 432 average length = 332
fDETECT	*a =* 0.12180127	0.71 h	0.90 min	0.88 min
	*b =* 8.2653E-06			
PPCpred	*a =* 88.742223	76.15 days	27.49 h	28.35 h
	*b =* 0.42263874			
Crysalis	*a =* 0.07497269	0.73 h	0.77 min	0.77 min
	*b =* 9.814E-05			
PredPPCrys	*a =* 204.206124	79.12 days	34.04 h	34.48 h
	*b =* 0.23944459			

The analysis covers the four methods that predict the four steps of the protein production and crystallization process. The second column shows coefficients of a linear fit into the measured values of the runtime and protein sequence length on the TESTsmall dataset, i.e., runtime = a*sequence_length + *b*. The total runtimes estimated with that linear fit for the proteins in the complete human proteome and from the benchmark dataset are listed in columns three and four, respectively. The right-most column shows the total runtime that was empirically measured on the TESTsmall dataset

### Comparative evaluation of predictive performance

We assessed fDETECT’s predictive performance using two test datasets, TESTlarge and TESTsmall. Both datasets share <25% similarity with the training dataset of fDETECT. The large dataset is used to compare with Crysalis [25], the only other equally fast method that also covers prediction of the four steps of the protein production and crystallization pipeline. We applied the TESTsmall dataset to compare with the other slower predictors including the two methods that predict multiple steps of the protein production and crystallization pipelines: PPCpred [23] and PredPPCrys [24], the fast Crysalis, and four representative webservers that focus solely on the prediction of the diffraction-quality crystallization: XtalPred [7, 38], CRYSTALP2 [39], XtalPred-RF [40] and TargetCrys [37].

Table 3 compares predictive performance measured with AUC, MCC, and accuracy for each of the four outcomes on the TESTlarge dataset. The predictive performance measured with AUC for the MF, PF and CR steps is higher than for the CF step for both fDETECT and Crysalis. The same trend was observed as part of the evaluation by the authors of the Crysalis method [25]. The values of AUC, MCC and accuracy are modest and this is expected given the intrinsic limitations of the inputs. The predictive models in this area consider only the intra-molecular aspects that are encoded in the protein sequence. Consequently, the achievable predictive performance is limited by the fact that the outcomes also depend on a number of inter-molecular factors such as the expression systems used, protein–protein and protein–precipitant interactions, buffer composition, use of specific tags, etc. Analysis of MCCs and accuracies reveals that fDETECT provides modestly higher values for the MF and CR steps and substantially higher values for the PF and CF steps when compared to Crysalis. However, these differences are statistically significantly (*p*-value <0.02). Moreover, the improvements in AUC that are offered by fDETECT are large and statistically significant for the PF step (*p*-value <0.001) and modest and statistically significant for the CR steps (*p*-value <0.001). The differences in AUC for the other two steps are not statistically significant (*p*-value >0.23). We conclude that fDETECT offers a modestly better predictive performance when compared to Crysalis, the only other similarly fast and similarly comprehensive method.

**Table 3:** Predictive performance on the TESTlarge dataset

	Predictors	Material production (MF)			Purification (PF)			Crystallization (CF)			Diffraction-quality crystallization (CR)		
		Average	±std	*p*-value	Average	±std	*p-value*	Average	±std	*p-value*	Average	±std	*p*-value
AUC	fDETECT	**0.63**	±0.05		**0.65**	±0.05		**0.54**	±0.05		**0.62**	±0.04	
	Crysalis	**0.62**	±0.05	0.269	0.58	±0.05	<0.001	**0.55**	±0.05	0.234	0.60	±0.04	<0.001
MCC	fDETECT	**0.11**	±0.07		**0.19**	±0.07		**0.12**	±0.09		**0.19**	±0.07	
	Crysalis	0.10	±0.08	0.011	0.10	±0.07	<0.001	0.03	±0.08	<0.001	0.16	±0.06	<0.001
Accuracy	fDETECT	**75.3**	±2.0		**74.2**	±2.3		**66.9**	±3.4		**59.7**	±3.3	
	Crysalis	74.8	±2.1	0.012	70.9	±2.3	<0.001	63.6	±2.9	<0.001	58.1	±3.2	<0.001

We report average AUC, MCC and accuracy and their corresponding standard deviations over 100 bootstrap tests (each test is based on 25% of randomly chosen proteins). Statistical significance of differences between fDETECT and Crysalis was measured with paired *t*-test; the measured values are normal, which we verified based on the Anderson-Darling test at 0.05 significance. The best results that are not significantly different with each other (*p*-value >0.05) for each outcome are given in bold font

**Fig. 3:**
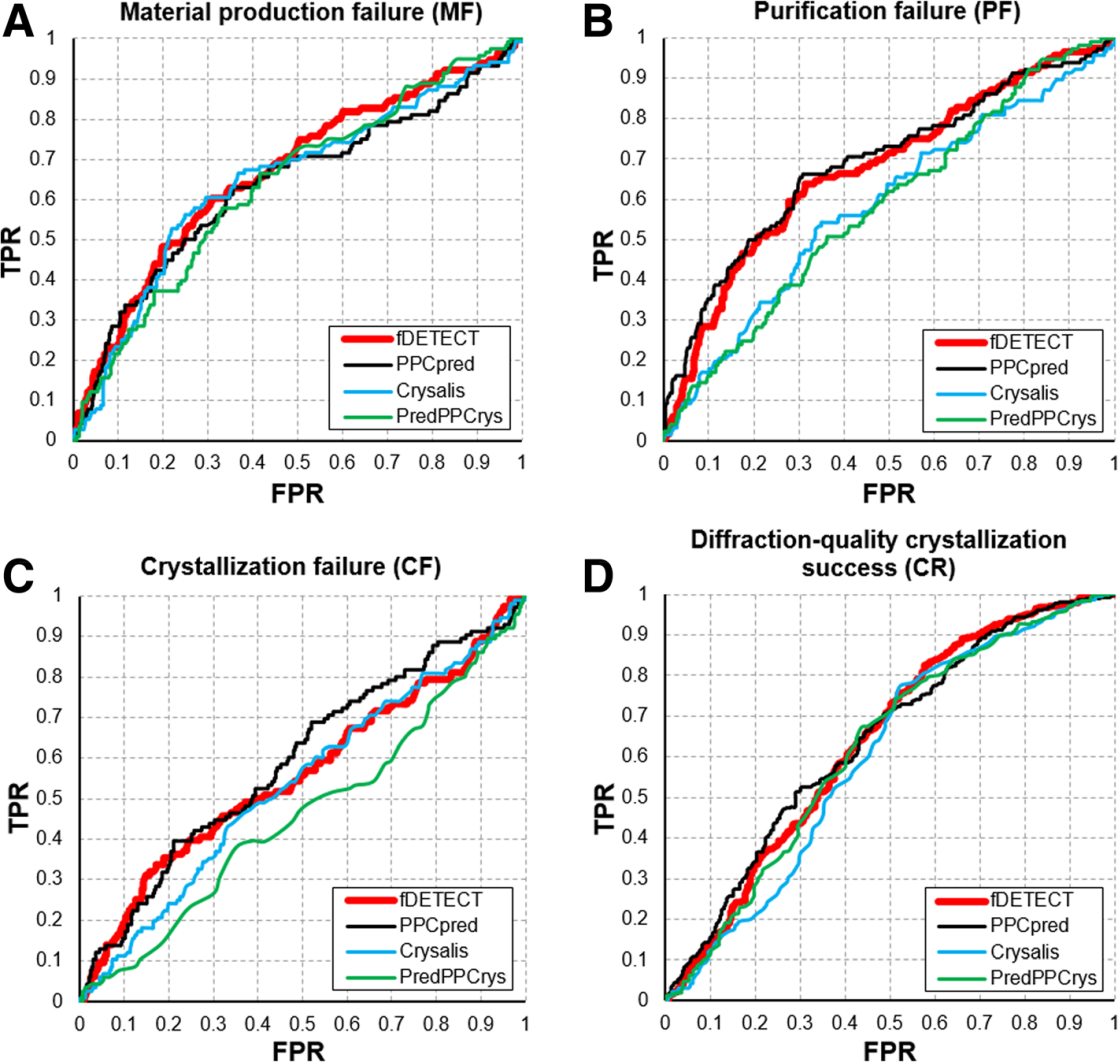
ROC curves for the four predictors of the four steps of the crystallization pipeline: failure of material production (panel **a**), failure to purify (panel **b**), failure to crystallize (panel **c**) and success to yield diffraction-quality crystals (panel **d**). The curves were computed on the TESTsmall dataset

Table 4 summarizes the results on the TESTsmall dataset. Similar to the results on the TESTlarge dataset, the values of AUC are the lowest for the CF step when compared to the other three steps. This is consistent across the four methods that predict multiple protein production and crystallization steps: fDETECT, PPCpred, Crysalis and PredPPCrys. Among these four predictors, AUC values of fDETECT and Crysalis are substantially higher than the AUCs of the other two methods for the prediction of the MF step; these differences are statistically significantly (*p*-value <0.01). PPCpred outperforms the other methods for the PF and CF steps, although the predictive performance for the CF step is overall modest; these improvements in AUC that are provided by PPCpred are statistically significant (*p*-value <0.001). Finally, fDETECT and PPCpred outperform Crysalis and are modestly better than PredPPCrys for the CR step; the increases in the AUCs by fDETECT and PPCpred compared to Crysalis and PredPPCrys are statistically significant (*p*-value <0.01). Figure 3 shows the corresponding ROC curves. It shows that the curves for fDETECT (red) and PPCpred (black) are close to each other and above the curves of the other two methods for the lower values of FPR < 0.2, i.e., when predictors generate modest numbers of false positives. That separation is relatively small for the MF and CR steps and much larger for the PF and CF steps.

**Table 4:** Predictive performance on the TESTsmall dataset

	Predictors	Material production (MF)			Purification (PF)			Crystallization (CF)			Diffraction-quality crystallization (CR)		
		Average	±std	*p*-value	Average	±std	*p-value*	Average	±std	*p-value*	Average	±std	*p*-value
AUC	fDETECT	**0.68**	±0.11		0.64	±0.11		0.55	±0.11		**0.64**	±0.07	
	PPCpred	0.64	±0.11	0.004	**0.67**	±0.12	<0.001	**0.60**	±0.12	<0.001	**0.66**	±0.08	0.054
	Crysalis	**0.67**	±0.11	0.392	0.59	±0.11	<0.001	0.56	±0.10	0.366	0.60	±0.08	<0.001
	PredPPCrys	0.62	±0.11	<0.001	0.59	±0.11	0.002	0.48	±0.12	<0.001	0.62	±0.08	0.001
	XtalPRed	NA			NA			NA			0.59	±0.09	<0.001
	XtalPred-RF	NA			NA			NA			**0.65**	±0.08	0.392
	TragetCrys	NA			NA			NA			**0.64**	±0.07	0.734
	CRYSTALP2	NA			NA			NA			**0.63**	±0.08	0.419
MCC	fDETECT	**0.21**	±0.17		0.15	±0.19		**0.16**	±0.18		0.20	±0.13	
	PPCpred	0.19	±0.20	0.039	**0.26**	±0.21	<0.001	**0.20**	±0.18	0.752	0.23	±0.15	0.253
	Crysalis	**0.20**	±0.18	0.115	0.11	±0.19	0.018	0.07	±0.16	<0.001	0.15	±0.15	0.005
	PredPPCrys	0.12	±0.17	<0.001	0.06	±0.18	<0.001	0.00	±0.19	<0.001	0.19	±0.15	0.314
	XtalPRed	NA			NA			NA			0.18	±0.18	0.580
	XtalPred-RF	NA			NA			NA			**0.24**	±0.14	0.039
	TragetCrys	NA			NA			NA			0.21	±0.12	0.889
	CRYSTALP2	NA			NA			NA			0.23	±0.14	0.297
Accuracy	fDETECT	**78.0**	±4.9		72.7	±6.2		**68.5**	±6.9		**59.9**	±6.7	
	PPCpred	77.4	±5.4	0.040	**76.2**	±6.7	<0.001	**69.9**	±6.6	0.770	**61.4**	±7.6	0.211
	Crysalis	77.7	±4.9	0.104	71.4	±6.0	0.018	65.1	±6.1	<0.001	57.5	±7.3	0.004
	PredPPCrys	75.6	±4.8	<0.001	70.0	±6.0	<0.001	61.9	±7.5	<0.001	**59.3**	±7.5	0.348
	XtalPRed	NA			NA			NA			**58.7**	±8.8	0.315
	XtalPred-RF	NA			NA			NA			**62.2**	±7.2	0.117
	TragetCrys	NA			NA			NA			**60.3**	±6.2	1.000
	CRYSTALP2	NA			NA			NA			**61.4**	±6.7	0.256

We report average AUC, MCC and accuracy and their corresponding standard deviations over 100 bootstrap tests (each test is based on 25% of randomly chosen proteins). Statistical significance of differences between fDETECT and each other method was measured with paired *t*-test; the measured values are normal, which we verified based on the Anderson-Darling test at 0.05 significance. The best results that are not significantly different with each other (*p*-value >0.05) for each outcome are given in bold font. NA means that a given method does not provide this type of prediction

Moreover, side-by-side comparison of AUC values of these four methods against the four methods that predict only the CR step (XtalPRed, XtalPred-RF, TragetCrys and CRYSTALP2) demonstrates that AUC of fDETECT is among a group of five methods that offer the highest and statistically similar predictive performance (*p*-value >0.05). The two methods that secure substantially lower AUCs for the CR step when compared to fDETECT are Crysalis and XtalPred; these differences are statistically significant (*p*-value <0.001). The difference in AUC between Crysalis and PredPPCrys is modest but statistically significant (*p*-value = 0.01). We observed a similar trend when considering MCC and accuracy measures. For instance, the highest accuracy for the MF step is achieved by fDETECT (*p*-value <0.05), for the PF step by PPCpred (*p*-value <0.001), and for the CF step by fDETECT and PPCpred (*p*-value <0.001). All methods except for Crysalis secure similar levels of accuracy for the CR step. The accuracy of Crysalis is modestly lower but this difference, when compared with fDETECT, is significant (*p*-value = 0.004). To sum up, fDETECT and PPCpred provide modest levels of predictive performance that are better than the results of the other methods for the PF and CF steps. Furthermore, most of the current methods, including fDETECT, are equally accurate for the prediction of the CR step.

Finally, we compared the fDETECT’s predictive performance on the test datasets with its performance based on the five-fold cross-validation on the training dataset. The MCCs for the MF, PF, CF and CR steps on the training datasets (average ± standard deviation based on the per test fold results) are 0.23 ± 0.02, 0.17 ± 0.04, 0.09 ± 0.06, and 0.33 ± 0.02, respectively. To compare, Table 3 (Table 4) lists the corresponding MCCs on the TESTlarge (TESTsmall) datasets as 0.11 ± 0.07 (0.21 ± 0.17), 0.19 ± 0.07 (0.15 ± 0.19), 0.12 ± 0.09 (0.16 ± 0.18), and 0.19 ± 0.07 (0.20 ± 0.13), respectively. We observed that MCCs for the first three outcomes are in good agreement while the predictive performance on the CR class is better on the training dataset. Analogous conclusions can be drawn from the AUC values. The fDETECT’s AUCs on the training dataset equal 0.65 ± 0.01 for the MF step, 0.68 ± 0.01 for the PF step, 0.62 ± 0.03 for the CF step, and 0.74 ± 0.01 for the CR step. To compare, the corresponding AUCs in Table 3 (Table 4) are 0.63 ± 0.05 (0.68 ± 0.11), 0.65 ± 0.05 (0.64 ± 0.11), 0.54 ± 0.05 (0.55 ± 0.11), and 0.62 ± 0.04 (0.64 ± 0.07), respectively. Again, they reveals similarly consistent levels of predictive performance between the training and test datasets.

## Discussion and conclusions

**Fig. 4:**
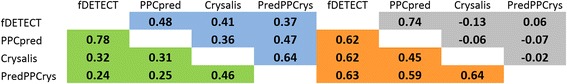
Correlation between propensities generated by fDETECT and the three other methods that cover multiple steps of the crystallization pipeline: PPCpred, PredPPCrys and Crysalis. We report the values of the Pearson correlation coefficient (PCC) for the each of the four steps: MF (on blue background), PF (green), CF (orange) and CF (grey)

Motivated by the lack of fast and accurate sequence-based predictors of propensity for protein production and structural determination [20], we delivered a new fDETECT method. fDETECT is available as a convenient and publically accessible webserver that addresses prediction of propensity for material production failure (MF), purification failure (PF), crystallization failure (CF) and successful diffraction-quality crystallization (CR). We conducted comprehensive empirical tests to compare runtime and predictive performance of fDETECT to the performance of the three existing methods that offer the same scope of predictions. The tests reveal that fDETECT generates predictions that are three orders of magnitude faster than PPCpred and PredPPCrys methods and similarly fast when compared to the Crysalis method. To put it into perspective, fDETECT and Crysalis can be used to predict the human proteome in under an hour, while the same prediction would take PPCpred and PredPPCrys close to 80 days. Our empirical tests show that fDETECT offers modest levels of predictive performance that are better that the predictive quality of the equally fast Crysalis and the slower PredPPCrys. The results also reveal that fDETECT complements the predictive performance of the substantially slower PPCpred. More specifically, fDETECT provides more accurate results for the MF step, PPCpred for the PF and CF steps, and both methods secure similar results for the CR step. Figure 4 show values of Pearson correlation coefficient (PCC) between the propensities generated by each pair of methods for the prediction of the four steps of the protein production and structural determination process. The results demonstrate that propensities produced by fDETECT and PPCpred share modest levels of PCC for the MF steps (0.48) and relatively high PCC for the other three steps (PCC ≥ 0.62). The propensities output by the other fast predictor, Crysalis, are highly correlated with the fDETECT’s propensities for the CR step (0.62), modestly correlated for the MF and PF steps (PCC ≤ 0.41) and lack correlation for the CF step (PCC = −0.13). Interestingly, PCCpred and fDETECT are the only two methods that have correlated outputs for the CF step. The correlations of the propensities for all other pairs of predictors are very low, likely because of the overall lower predictive performance for this step. We also noted the relatively high PCC values for the CR step across all pairs of the four predictors (results on the orange background in Fig. 4). Finally, when compared to the four representative webservers that predict propensity for the diffraction-quality crystallization (XtalPRed, XtalPred-RF, TragetCrys and CRYSTALP2), we demonstrated that fDETECT offers equivalent predictive quality while it has the advantage of providing the other three predictions at a minimal computational cost.

We observed that the overall predictive performance of the most accurate methods is relatively modest, with AUCs in the 0.60 to 0.67 range and MCC between 0.20 and 0.26 (Table 4). As we discussed above, one of the most likely reasons is the limited scope of the information that can be extracted from the input protein sequence. This information does not include some of relevant details of the crystallization process. Another contributing factor is the fact that the source data were collected from across many structural genomics centers that may use different production and crystallization protocols for the same proteins. This may ultimately adversely affect the quality of the experimentally assigned outcomes. However, models with even such modest predictive quality are still practical. To provide context, the reported success rates to produce diffraction-quality crystals are below 10% [3, 4]. A recent post-hoc study shows that use of the tools that we consider in this article leads to a substantial improvement in the quality of the selection of proteins for structure determination when compared with an ad hoc target selection [30]. Another point to support this claim of practicality is the fact that these methods found interest in the community. For instance, some of these predictors are utilized directly by the structural genomics centers, including MCSG-Z score [44] by the Midwest Center for Structural Genomics and XtalPred by the Joint Center for Structural Genomics. Other methods that are not associated with structural genomics centers report relatively heavy use. As an example, our PPCpred method that is running continually as a webserver since 2011 has processed requests from 2816 unique users coming from 60 countries and 515 cities [source: Google Analytics as of Sept 7, 2017].

The predictive performance that we reported is lower and arguably more robust than the results that were published by the authors of PredPPCrys [24] and Crysalis [25]. The main reason for these discrepancies is that the other authors have used different proportions of proteins at different steps of the protein production and crystallization pipeline. In particular, about 71% of the proteins in their test datasets were assigned to the MF step and only 1% to the CF step, which coincidentally is the most difficult to predict. Moreover, in the original articles Crysalis and PPCpred were tested on the test datasets that shared much higher sequence similarity with their training dataset, 40% for Crysalis and unlimited for PPCpred. In contrast, our test datasets share up to 25% similarity with the training proteins.

To summarize, we anticipate that the fDETECT webserver will become a popular tool to support protein production efforts and X-ray crystallography-based structure determination, especially for the analysis of large sets of proteins.

## Abbreviations

AUC: Areas Under the receiver operating characteristic Curve
CF: Crystallization Failure
CR: diffraction-quality CRystallization
fDETECT: fast Determination of Eligibility of TargEts for CrysTallization
MCC: Matthews Correlation Coefficient
MF: Material production Failure
PDB: Protein Data Bank
PF: Purification Failure
